# Click communication in wild harbour porpoises (*Phocoena phocoena*)

**DOI:** 10.1038/s41598-018-28022-8

**Published:** 2018-06-26

**Authors:** P. M. Sørensen, D. M. Wisniewska, F. H. Jensen, M. Johnson, J. Teilmann, P. T. Madsen

**Affiliations:** 1 Zoophysiology, Department of Bioscience, Aarhus University, C.F. Moellers Allé 3, DK-8000 Aarhus C, Denmark; 2 Hopkins Marine Station, Stanford University, 120 Ocean View Blvd, Pacific Grove, CA 93950 USA; 3 Aarhus Institute of Advanced Studies, Aarhus University, DK, Høegh-Guldbergs Gade 6b, 8000 Aarhus C, Denmark; 4 Sea Mammal Research Unit, Scottish Oceans Institute, University of St. Andrews, St. Andrews, Fife, KY16 8LB United Kingdom; 5 Marine Mammal Research, Department of Bioscience, Aarhus University, Frederiksborgvej 399, 4000 Roskilde, Denmark

## Abstract

Social delphinids employ a vocal repertoire of clicks for echolocation and whistles for communication. Conversely, the less social and acoustically cryptic harbour porpoises (*Phocoena phocoena*) only produce narrow-band high-frequency (NBHF) clicks with properties that appear poorly suited for communication. Nevertheless, these small odontocetes likely mediate social interactions, such as mate choice and mother-calf contact, with sound. Here, we deployed six tags (DTAG3) on wild porpoises in Danish waters for a total of 96 hours to investigate if the patterns and use of stereotyped NBHF click trains are consistent with a communication function. We show that wild porpoises produce frequent (up to 27 $\bullet$ min^−1^), high-repetition rate click series with repetition rates and output levels different from those of foraging buzzes. These sounds are produced in bouts and frequently co-occur with emission of similar sounds by nearby conspecifics, audible on the tags for >10% of the time. These results suggest that social interactions are more important to this species than their limited social encounters at the surface may indicate and that these interactions are mediated by at least two broad categories of calls composed of short, high-repetition rate click trains that may encode information via the repetition rate of their stereotyped NBHF clicks.

## Introduction

Animals exchange information and coordinate their behaviour via communication mediated by a variety of sensory modalities. In the marine environment, visual cues are only available over short distances and chemical signals propagate slowly^1^. Sound, on the other hand, propagates rapidly and over long ranges, giving a selective advantage to the use of acoustics for navigation, foraging and communication. In general, low frequency sounds radiate with low directionality and suffer little from absorption whereas higher frequency sounds from the same source will be more directional and experience greater absorption^2^. This may explain why many toothed whales employ a rich vocal repertoire of high-frequency clicks primarily for echolocation^3^ and lower frequency tonal sounds for communication^4,5^.

A well-studied example of a species with a diverse vocal repertoire is the bottlenose dolphin (*Tursiops spp*.) that lives in complex fission-fusion societies^6^ where decade-long social recognition of conspecifics^7^ allows for differentiated social relationships including multiple levels of alliance formation^8,9^. It has been hypothesised that such social complexity co-evolved with increasingly complex communication signals^10^, such as individually specific signature whistles^11^ that allow for long-term recognition^12^, group-^13^ and mother-calf cohesion^14,15^ and referential labeling of conspecifics^16^. In contrast, other toothed whale species, such as Kogias^17^, the franciscana river dolphin^18^, delphinids of the genera *Cephalorhyncus* and *Lagenorhyncus*^19,20^ and porpoises, live in smaller groups where acoustically mediated social interactions with conspecifics may be less common. This notion is supported by the fact that they do not produce whistles, but only narrow-band high-frequency (NBHF) clicks^21^. Such convergent evolution on a vocal repertoire consisting of only NBHF clicks has led to the hypothesis that these species produce clicks well above the frequency range of best hearing sensitivity in killer whales^22,23^ to reduce predation and harrasment by such large delphinids^24–27^. Thus, in contrast to other odontocetes aggregating in large groups as a predator defence mechanism^25^, NBHF species may have evolved acoustic crypsis to avoid their predators.

While adopting an acoustic crypsis strategy may help NBHF species avoid predators, it involves potential socioecological and functional tradeoffs. The high-frequency and directional nature of NBHF clicks results in a small active space for conspecifics to detect emitted signals, thus potentially limiting social interactions^28^. Furthermore, if porpoises use NBHF signals for communication as well as for echolocation, conspecifics need to be able to differentiate communication signals from foraging sounds to decrease signal ambiguity. Despite these challenges porpoises nonetheless depend on interactions with conspecifics for critical behaviours such as mating and parental care, and given the importance of sound for mediating such processes in other cetaceans, it would seem likely that NBHF species also rely on acoustic communication.

A few studies have attempted to assess the potential for acoustic communication in NBHF species. For Hector’s dolphins, it has been proposed that information is conveyed through the timing of click emissions, although this is based on limited knowledge on the context of call production^29^. The same was suggested in a study on captive harbour porpoises^30^, where playbacks of high-repetition rate click trains elicited a flight response. In two other independent studies on captive harbour porpoises distinct, high-repetition rate click trains were associated with specific behaviours^28,31^. Together, these observations suggest that captive harbour porpoises use specific click trains to communicate, however it remains unknown how often wild porpoises employ acoustic communication given their apparently infrequent social encounters.

To address that data gap, we use acoustic and movement recording DTAGs to investigate the use of click trains by wild harbour porpoises for the purpose of communication, and to quantify the degree to which individuals are in acoustic contact with each other. Using tags on six wild porpoises, we show that they produce a large number of click trains with repetition rate patterns distinct from foraging buzzes but similar to the communication calls emitted by captive harbour porpoises^28^. This suggests that social interactions are more important to this species than their limited social encounters observed at the surface may indicate, and that these interactions are mediated via acoustic information transmitted through the repetition rate patterns of NBHF clicks.

## Results

Tags deployed on six wild harbour porpoises provided a total of 96 hours of recordings (Table 1). Four of the porpoises were found in the pound nets alone, whereas the two females with calves were found with the calf either in or just outside the pound net (Table 1).

**Table 1:** Tag deployment and data summary.

animal ID	hp12_272a	hp12_293a	hp13_102a	hp14_226b	hp15_218a	hp16_264a
date and location of deployment	28/09/2012 56°10.14′N 10°31.53′E (Begtrup Vig)	19/10/2012 56°10.14′N 10°31.53′E (Begtrup Vig)	12/04/2013 55°22.40′N 11°08.14′E (Korsør)	14/08/2014 56°10.14′N 10°31.53′E (Begtrup Vig)	06/08/2015 56°10.14′N 10°31.53′E (Begtrup Vig)	20/09/2016 55°22.40′N 11°08.14′E (Korsør)
animal age and sex	Juvenile ♀	Adult ♀ and a calf (calf found outside the net)	Juvenile ♂	Juvenile ♂	Adult ♀	Adult ♀ and a calf (calf found inside the net)
standard length (cm)	122	163	114	126	156	163
recording duration (hours)	21.9	17.7	24	20	2.5	12
tagged animal calls (n)	250	918	787	1004	88	657
call rate (calls · h^−1^)	11	52	33	50	35.2	55
tagged animal positive call minutes (% of minutes that include calls)	7.7	31.5	8.5	19.1	19.0	27.5
maximum interval between conspecific vocalisations (min)	180	22	95	168	31	35
conspecific occurrence (% of minutes with audible presence of any conspecifics vocalisations)	17.8	58.8	35.8	10.5	9.9	54.0
conspecific call occurrence (% of minutes that include conspecfic calls)	2.3	34.4	3.6	2.3	1.7	18.5

### Porpoise diving and vocal behaviour

**Figure 1:**
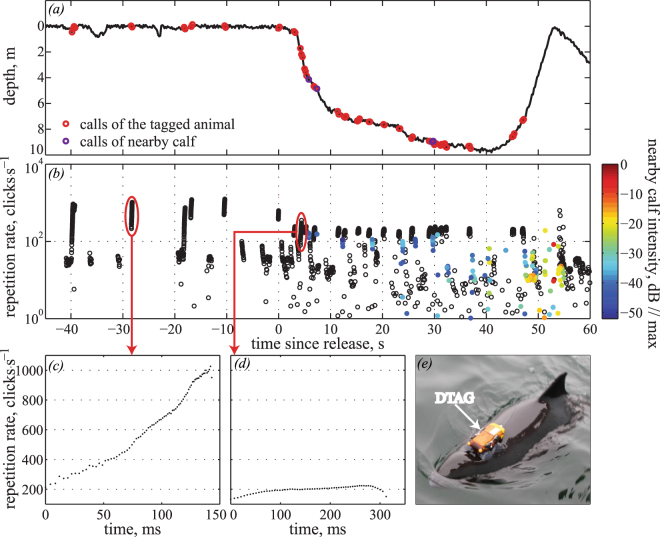
Example dive profile and calls of a tagged adult female (hp12_293a), associated with a calf, at the time of release. (**a**) Dive profile of porpoise hp12_293a from the time of tagging to 60 seconds after release from the boat. (**b**) Repetition rate of clicks emitted by this porpoise (black circles) and its calf (filled coloured circles). Colour coding indicates relative received level of the calf’s clicks. With time after release the received level of clicks emitted by the calf increases by up to 50 dB. Examples of a high–repetition rate call emitted by hp12_293a before release (**c**) and a call of lower repetition rate, emitted by the animal just after release from the boat (**d**). (**e**) A porpoise tagged with a DTAG3 data logger.

**Figure 2:**
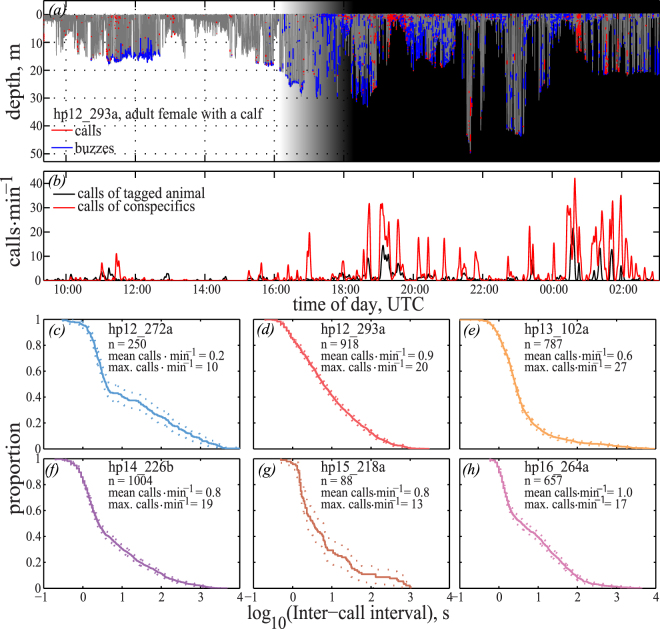
Calling behaviour and call rates of wild harbour porpoises. Calling behaviour of an adult female (hp12_293a) tagged in the proximity of her calf. (**a**) The full dive profile, with individual buzzes in blue and calls in red. The shaded area represents twilight (grey) and night (black). (**b**) Minute-wise call rate for the tagged animal (black) and conspecifics (red). (**c**–**h**) Survivor functions representing the time elapsed between calls for each animal.

Throughout the recordings, all porpoises produced NBHF clicks almost continuously with the first clicks emitted within minutes after release. All vocalisations were composed of NBHF clicks. In addition to the easily recognisable buzzes, all tagged animals also emitted high-repetition rate click trains that both sounded and appeared in spectrograms to be distinct from buzzes^32^. One individual (hp12_293a), an adult female associated with a calf, emitted a series of click trains at a repetition rate of more than 1000 clicks · s^−1^ (inter-click interval (ICI) < 1 ms) (Fig. 1c) in air shortly after tag attachment on-board the boat (Fig. 1). At release, she continued vocalising (Fig. 1), emitting click sequences with a repetition rate of up to 250 clicks · s^−1^ (ICI < 4 ms) (Fig. 1d). Additionally, just seconds after she was released, similar click trains from her calf were recorded on the tag (Fig. 1b). The amplitude of the clicks from the calf increased with time from release indicating that the mother and calf were approaching each other until both animals suddenly ceased calling after approximately 50 seconds (Fig. 1b). This female, as well as all other tagged animals, went on to produce numerous click trains that were judged to be calls throughout the tag deployment (see Supplementary Material, Fig. 2). Calls were produced at a high rate for all individuals (Fig. 2c–h) and were similar to the social calls that have been described for captive porpoises^28^. Careful auditing revealed 88 calls recorded from the individual with the shortest tag deployment (Fig. 2g, Table 1) and more than 1000 calls emitted throughout one of the longer deployments (Fig. 2f, Table 1). Calls were emitted at a mean rate ranging from 0.2–1.0 call · min^−1^ and often in call bouts with rates of up to 27 calls · min^−1^ (Fig. 2, Table 1) in bout intervals of five-six minutes.

Calls from the tagged animal often co-occurred with similar calls from conspecifics and the maximum time without the acoustic presence of conspecifics ranged between 22 and 180 minutes (Table 1). Individual recordings had a high percentage of conspecific positive minutes between 9.9% and 58.8% of all one-minute intervals of the total recording time (Table 1). For the two females accompanied by calves and for one juvenile male, the call rate for each tagged animal averaged over six minute bins was significantly correlated with the rate of calls from nearby conspecifics (hp12_293a: p < 0.0002, hp13_102a: p < 0.0002, hp16_264a: p < 0.0322 at 5000 permutations) (Fig. 2b).

### Validating the functional distinction between buzzes and calls

In total, 14,087 high-repetition rate click trains were identified, of which 74% (N = 10,383) were marked as buzzes and 26% (N = 3,704) as possible calls in the initial evaluation. A subset of 528 possible calls and 528 buzzes (e.g. Supplementary Fig. S1) from all animals were presented to five independent evaluators to test if the distinction between buzzes and possible calls was in line with the auditing. 91% of the 528 (88 per animal) high-repetition rate click trains initially marked as possible calls were determined by the evaluators to be distinct from buzzes as neither prey echoes, nor jerks were present, whereas 97% of all 528 (88 per animal) high-repetition rate click trains originally marked as buzzes were assessed to be associated with prey echoes and/or jerks (See Supplementary Fig. S2). The Fleiss’s Kappa inter-rater agreement coefficient was 0.877 (C.I. (95%) = 0.872 to 0.882)^33^. Based on this evaluation, we accepted all possible calls identified in the preliminary analysis as actual calls with a communication function and therefore functionally distinct from buzzes. The few sounds that may be misclassified between these two categories will have little impact on the results.

### Acoustic properties of calls

Acoustic parameters of individual clicks within calls and regular clicks were calculated to test if signals judged to be used for communication are different from those of NBHF clicks used for echolocation. Supplementary Figure S3 shows a click emitted in a click train classified as a call and a click emitted during echolocation by a tagged animal and a nearby conspecific. Despite their use for two different purposes, the two clicks contain energy within the same range of frequencies. Furthermore, centroid frequency, −10-dB bandwidth and −10-dB duration estimates overlap for clicks emitted in calls and during regular echolocation, respectively (Table 2). This indicates that the same NBHF clicks are used for both echolocation and communication.

**Table 2:** Centroid frequency, −10-dB bandwidth and −10-dB duration of clicks in calls and regular echolocation clicks.

	Call clicks			Regular clicks		
	10^th^	median	90^th^	10^th^	median	90^th^
centroid frequency (kHz)	166	192	211	173	198	215
−10-dB BW (kHz)	44	80	112	55	86	112
−10-dB duration (µsec)	58	132	200	62	136	206

**Figure 3:**
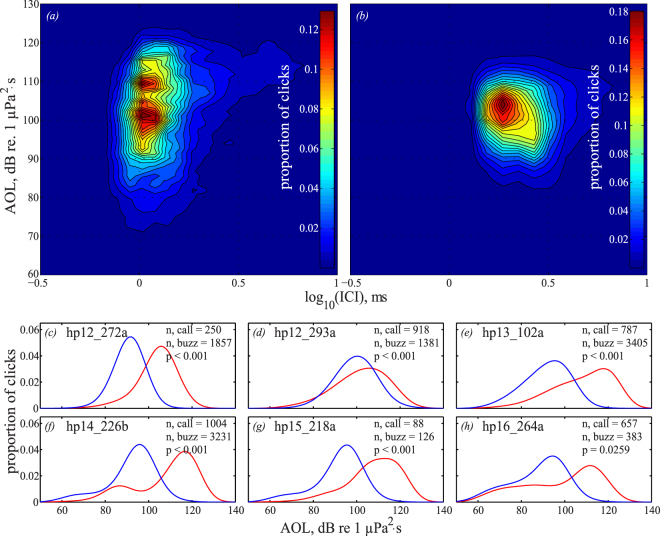
Differences in apparent output level (AOL) of clicks emitted in calls and buzzes. Kernel density estimation of the AOL as a function of inter-click-interval (ICI) with a bin-size of N = 64 for (**a**) all clicks contained in calls and (**b**) all clicks contained in buzzes emitted by animal hp12_293a. Blue represents low density of clicks, whereas red represents high density of clicks. (**c**–**h**) The probability density function of AOL of call clicks (red) and buzz clicks (blue) with a bandwidth of 1 dB for all six individuals. Given for each individual is the number (n) of buzzes and calls, as well as the p-value of the Kruskal-Wallis test investigating the individual difference in AOL of calls and buzzes.

The mean apparent output level (AOL^34^) of buzzes and calls for all six individuals, respectively, ranged between 81 and 101 dB re 1µPa^2^ ·s and 86 and 111 dB re 1µPa^2^ ·s (Fig. 3, Supplementary Table S1). We averaged the AOL (dB re 1µPa^2^ ·s) of all clicks in three categories: calls, buzzes and regular clicks (10,000 randomly selected), for each individual and compared these categories by subtracting the mean for each individual to account for individual differences in sound output and tag placement. The mean AOL of all regular echolocation clicks (111 dB re 1 µPa2 ·s) was significantly higher than both the mean AOL of all call clicks (102 dB re 1µPa^2^ ·s; kruskal-wallis test; p = 0.0065) and all buzz clicks (92 dB re 1µPa^2^ ·s; kruskal-wallis test; p = 0.0039). Additionally, the mean AOL of all call clicks for each individual was significantly higher than that of buzz clicks (kruskal-wallis; p = 0.0039).

**Figure 4:**
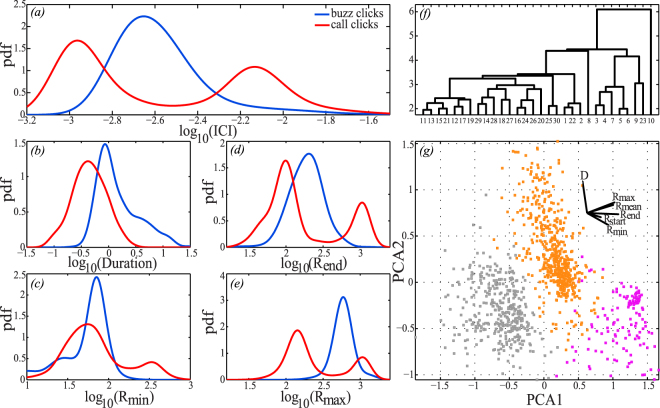
Investigation of how porpoises may be able to distinguish calls from buzzes. (**a**) Power density function (pdf) of inter-click-intervals for individual clicks separated out into buzz clicks (n = 394,924, blue) and call clicks (n = 67,099, red) (Kernel density estimation, Gaussian window of 0.1-unit length). All clicks have been extracted from the subset of the 88 possible calls and 88 possible buzzes per individual (the evaluator dataset, see Supplementary Material). (**b**–**e**) Distribution of four acoustic parameters - duration (log_10_ to seconds), minRR, endRR and maxRR (log_10_ to clicks · s^−1^) - for the two types of high-repetition rate click trains. StartRR and endRR are defined as the median ICI between the first six clicks and the last six clicks in a click train, respectively. (**f**) Dendrogram presenting unsupervised hierarchical cluster analysis based on the same six acoustic parameters as in (**b**–**e**). All parameters have been log- and z-transformed to represent each parameter by equal weight. The dendrogram is based on Euclidian distances and formed using an average linkage method. (**g**) K-means clustering based on the six log-transformed acoustic parameters, presented by a standard PCA. PCA1 + 2 only account for 83% of the variation, hence 17% of the variation is not visualised in the PCA plot (see also Table 3).

Individual ICIs were compared between calls and buzzes. Whereas buzz ICIs were relatively similar across individuals, the mean call ICI was more variable across individuals (See Supplementary Table S1). This pattern was particularly pronounced when the ICIs of individual clicks within buzzes and calls were compared (Fig. 4a) revealing a generally little overlap between the ICIs (Fig. 4).

### Clustering of call types

K-means clustering assigned the subset of 528 calls and 528 buzzes to three clusters consisting of low-repetition rate calls (100–600 clicks ·s^−1^), high-repetition rate calls (800 ->1000 clicks ·s^−1^) and buzzes (grey, purple and orange, respectively, Fig. 4g; Table 3). Three clusters were also found when performing an unsupervised hierarchical clustering analysis based on the same six parameters (dendrogram, Fig. 4f). PCA1 + 2 accounted for 83% of the variation, hence ~17% of the variation is not visualised in Fig. 4g (Table 3). The variables loading highest on PCA1 were end repetition rate (RR) (Fig. 4d) and maxRR (Fig. 4e), while PCA2 was mostly influenced by duration (Fig. 4b) and minRR (Fig. 4c, Table 3). All four parameters individually showed a distinction between buzzes and calls (Fig. 4b–e).

**Table 3:** Results of the K-means clustering analysis (see also Fig. 4).

	PCA1		PCA2	PCA3		PCA4	PCA5		PCA6
Duration	−0.08		0.86	0.44		−0.09	0.23		0.00
MeanRR	0.47		0.22	−0.13		0.07	−0.40		0.74
MinRR	0.39		−0.33	0.73		−0.41	−0.17		−0.07
MaxRR	0.49		0.29	0.26		0.06	−0.40		−0.67
StartRR	0.25		−0.12	0.35		0.87	0.20		−0.04
EndRR	0.56		−0.03	−0.26		−0.23	0.75		0.03
**Cumulative % explained**	**52**.**30**		**83**.**00**	**90**.**81**		**96**.**07**	**99**.**66**		**100**.**00**
**Clustering categories**	**Buzz**			**Low RR calls**			**High RR calls**		
**Originally marked as**	**Call**	**Buzz**	**Total**	**Call**	**Buzz**	**Total**	**Call**	**Buzz**	**Total**
hp12_272a	1	87	88	87	1	88	0	0	0
hp12_293a	15	82	97	19	5	24	54	1	55
hp13_103a	1	84	85	87	0	87	0	4	4
hp14_226b	1	87	88	87	1	88	0	0	0
hp15_218a	10	84	94	8	3	11	70	1	71
hp16_264a	12	86	98	73	2	75	3	0	3

Loading of six acoustic parameters on the principal components, together with the cumulative variation explained by the components (top), resulting in the clustering of three categories; buzzes, low-repetition rate calls and high-repetition rate calls.

High-repetition rate calls were emitted by three individuals, but with two individuals producing the majority of the calls (Table 3). Low-repetition rate calls were emitted by all individuals (Table 3).

## Discussion

Harbour porpoises have a vocal repertoire consisting solely of high-frequency, short-range and narrow-band (NBHF) clicks that are well-suited for echolocation, but that appear unsuited for communication. This has been hypothesised to indicate a dependence on acoustic crypsis as a predator defence mechanism^25^, in which low-frequency calls are sacrificed to reduce detection probability. Indeed, while most delphinids mediate complex social interactions by using low-frequency tonal sounds, harbour porpoises appear to have a more solitary lifestyle with infrequent social interactions. However, while the average group size in the field is small, porpoises are at times seen in groups of two-three individuals^35^ and they may well benefit from acoustic communication during these social encounters as well as for locating mates and rearing offspring. A few studies have shown that captive porpoises do seem to communicate by emitting stereotyped click trains of NBHF clicks^28,30,31^, but it is unknown to what degree porpoises in the wild produce such sounds to mediate social interactions, nor how often they produce them. To address that lack of understanding we here deployed DTAG3s on six wild harbour porpoises to investigate the level of acoustic contact between wild porpoises and to address whether they use NBHF click trains for communication similar to those reported from animals in captivity.

Surprisingly, given the hypothesised infrequent sociality of phocoenids, we show that such calls occur frequently, in dense bouts with repetition rates of up to 27 calls∙min^−1^ and are separated by relatively short silent periods (Fig. 2, Table 1), suggesting that porpoises invest considerable time and energy in social communication. Such high call rates are not limited to the two mother-calf pairs; in fact, the highest repetition rates were found in a single tagged animal (Fig. 2e). In addition to the high call rates of the tagged animals, calls and echolocation clicks from conspecifics were frequently detected in the recordings. Conspecific echolocation clicks were detected 10–36% of the time in recordings from animals that were alone when tagged (Table 1) and from 54–59% of the time for the two tagged mothers accompanied by a dependent calf (Table 1). Additionally, for three of the tagged animals, including the two mother-calf pairs we observed a significant correlation between tagged animal call rate and conspecific call rate. For mother-calf pairs in particular, this may imply that such calls are important for maintaining cohesion between closely associated animals. The time between social encounters was short for all tagged animals (Table 1), but despite this relatively high incidence of conspecific vocalisations, it is likely that the presence of conspecific calls and clicks available to the tagged porpoises was even higher than these estimates, as the sensitivity of the porpoise auditory system exceeds that of our recording equipment^36^. Thus, the conservative estimates of conspecific encounters reported here implies that porpoises, at least in the shallow inner Danish waters, often come within audible range of conspecifics, despite a lack of specialised long-range acoustic signals. Additionally, although very little is known about porpoise social structure, these data suggest that they frequently encounter conspecifics in the wild, providing socialising, mating opportunities or even the possibility of cooperative foraging and hence the likely need to mediate such interactions with sound.

Porpoises employ high-frequency and relatively narrow-band signals to achieve efficient biosonar operation requiring a high sound source directionality to produce detectable echoes from small targets and to reduce the effect of clutter^3,17^. However, co-opting this signal for communication results in a small, directional active space for individual calls^28^ compared to delphinid whistles, which are much more suitable for broadcasting communication^37^. Porpoises may partly compensate for the high directionality of their calls by increasing source levels or by widening the radiation pattern from their melons^38^ judging from the higher AOL of calls compared to buzzes from tagged porpoises (Fig. 3); both will render a larger active space than for buzzes. A less directional transmission of the call energy would result in a shorter on-axis detection range, but a better detection range for conspecifics outside of the main sound beam and overall a larger active space. However, the limited degree to which porpoises appear to modulate directionality^38,39^ is unlikely to fully explain the observed change in AOL, suggesting that the increase in AOL during call emission may be due to a combination of both directionality and source level changes as compared to buzzes. In either case, the resulting increase of active space is ultimately limited by the high absorption at 130 kHz, and it is unlikely that porpoises will be able to hear conspecifics beyond 1000 meters^28^ even under the most favourable conditions. In that light, the high call rates (Fig. 2) and short periods of silence between calls may indicate that porpoises compensate for a small active space through redundancy, repeatedly calling out in different directions to advertise their position or to establish contact with conspecifics.

In addition to the reduced active space, another price to pay when communicating with NBHF clicks is the risk of signal ambiguity when the individual clicks used for communication are indistinguishable from those used for echolocation in navigation or foraging. To circumvent that problem, it has been proposed that porpoises encode information in the click repetition rate of their calls^28,29,31^. If so, the ICI of calls should be in some way distinct from those of buzzes despite a potentially large variation in ICI adjustment during pursuit and capture of diverse prey throughout the water column^32^. Here we find that the mean ICI of buzzes was relatively constant, despite highly variable feeding conditions (See Supplementary Table S1, Fig. 4a), and similar to the ICI of buzzes reported for harbour porpoises in captivity^40,41^. In contrast, we find that calls differ from buzzes in both repetition rate and duration (Fig. 4b–g). Duration alone will most likely not enable distinction between calls and buzzes as the duration of buzzes varies with prey behaviour^32,41^ and thus there is significant overlap between the duration of calls and buzzes (Fig. 4b). However, since echolocating animals depend on auditory estimation of very small time delays for echolocation, the temporal differences in the repetition rate of buzzes and calls may well be perceived by listening conspecifics. Intriguingly, calls are not uniformly emitted with a lower ICI than buzzes – rather, it seems that porpoises avoid communicating at click rates that are used primarily during buzzing but that they may produce both lower- and higher-repetition rate calls (Fig. 4f,g). While all individuals emitted calls belonging to the low-repetition rate category, high-repetition rate calls were only produced by three, and primarily two, individuals (Table 3). Some of the high-repetition rate calls were emitted at a click rate of up to and above 1000 clicks·s^−1^ (Figs 1a,c and 4a) with a similar pattern to the calls that have been associated with aggressive interactions^28^. However, such calls were also emitted by several animals while they were being handled on the boat before release (Fig. 1a–c) in a similar manner to how bottlenose dolphins under the same settings emit signature whistles^42^. Thus, it may be speculated that some of these high-repetition rate calls either serve an aggressive function or perhaps a function similar to that of signature whistles in mediating cohesion between individuals.

If information in calls is encoded in the pattern of click intervals, the questions of whether and how porpoises can decode information at such high repetition rates remains open. Both their auditory envelope following response^43^ and the fact that porpoises display acute vocal-motor control during target interception at ICIs around 2–3 ms^38^ suggest that porpoises may be able to extract modulation information at very high click repetition rates. However, it remains to be tested what information is relayed through the calls and in what behavioural context different calls are used. Our results suggest that porpoises use at least two broad call categories and that individuals differ in how frequently they employ these calls. Carefully designed playback^44^ or interactive playback studies^45^ would enable testing of hypotheses about the function of such call categories. It is likely that the clustering employed here is too simplistic and that porpoises are able to relay detailed information in the click patterns of their calls that mediate a range of social interactions.

Here we show that harbour porpoises in the wild frequently are within audible range of conspecifics and that they produce a large number of high-repetition rate calls in dense bouts. The high call rates may help harbour porpoises to overcome some of the challenges in communicating with high-frequency, directional NBHF clicks that have properties well-suited for echolocation but are less suited for social communication. The potentially small active space of these high-frequency calls is also partly ameliorated by emitting calls at higher AOLs compared to foraging buzzes, showing that porpoises use a higher source level and/or decrease their transmission directivity when emitting calls. While individual clicks emitted during a call have the same spectral properties as regular echolocation clicks, conspecifics may discriminate calls from foraging buzzes based on call duration and click repetition rates. Collectively, these findings suggest that porpoises to a greater extent than previously assumed are in frequent contact with conspecifics and that their social encounters may be mediated by information conveyed by changing the repetition rates of the same stereotyped signals they use for echolocation, allowing porpoises to communicate acoustically while avoiding acoustic eavesdropping by large delphinids.

## Methods

### Study animals and tag deployment

Data were collected over a span of four years, from September 2012 to September 2016 where porpoises incidentally caught alive in pound nets upon their release were equipped with a high-resolution sound and movement recording tag with four silicone suction cups (DTAG^46^; see www.soundtags.org). The tagging was approved and carried out in accordance to relevant guidelines and regulations by the Danish Welfare Division (Ministry of Justice, 2010-561-1801 and 2015-15-0201-00549) and carried out under the permission from the Danish Forest and Nature Agency (NST-3446-0016).

The tag recorded 16-bit stereo sound continuously at a sample rate of 500 kHz, with a cliplevel of 179 dB re 1µPa and a flat (±2 dB) frequency response between 0.5 and 150 kHz. The tag also included a pressure sensor, tri-axial accelerometers and magnetometers sampled synchronously at rates between 250 Hz and 625 Hz, 16-bit. Accelerometer signals were filtered with a one-pole analogue low-pass filter with a −3 dB cut-off frequency of 50 Hz before digitizing. The animals were tagged within 24 hours of being discovered in the nets, and handling time during release and tagging ranged from three to 15 minutes. Furthermore, animals were not followed post-release. One tag (hp16_264a) was programmed to detach after 12 hours and remaining tags released unaided after 2.5 to 24 hours. The tags then floated to the surface, where they were retrieved using a combination of Argos satellite telemetry and/or aerial and boat-based tracking of their VHF beacons using yagi antennas and R1000 VHF receivers (Communications Specialists, Inc.).

### Data analysis

#### Data extraction

Data analyses were performed with custom-written scripts in Matlab R2013b (The Mathworks, Inc.). Acoustic data were first processed by visualisation and expert listening following established procedures^47^. Amplitude envelopes and spectrograms (Hamming window, FFT: 512, 50% overlap) of successive five-second segments of the recording were displayed along with a synchronised dive profile with the possibility of playing the audio simultaneously to aid identification of sounds. All vocalisations (only composed of NBHF clicks), as well as other sounds picked up by the tag (e.g. breaths, vessels, surface splashes) were marked, and their start cues and durations were saved. Vocalisations of the tagged animal were easily distinguished from those of conspecific individuals based on the presence of both low- (that could be heard during auditing) and high-frequency components of the signal when recorded off-axis as the signal bypasses the melon^48,49^ compared to the narrow band nature of non-focal clicks^32^. Because of the low frequency components in vocalisations from tagged animals these are audible in the sound recordings. Furthermore, the intensity of clicks in a click train produced by a conspecific are often less consistent compared to the clicks of the tagged animal, due to the varying spatial orientation of the conspecific sound source and the tag. Both tagged animal and conspecific vocalisations were marked during auditing. All audits were verified by a second analyst before being used further.

To detect backwards sliding of the tag, with or without an associated change in orientation, sound pressure levels of consecutive respirations were examined to reveal any systematic decrease. In case the tag was found to have moved, all subsequent click trains of interest were excluded from further analysis, to avoid biasing the estimates of apparent output level.

The subsequent analysis centred on two types of high-repetition rate (ICI < 15 ms^32^) click sequences, with characteristics similar to those described in the literature for foraging buzzes^32,41^ or possible communication calls^28,29^. Buzzes were generally easily recognisable throughout the recordings as they coincided with high flow noise indicative of a chase or strike at prey and most often were preceded by regular echolocation clicks^32^. However, some high-repetition rate click trains did not match these criteria and sounded distinctly different from presumed foraging buzzes and were labelled as possible calls. This initial tentative classification of high-repetition rate click trains was later re-evaluated in several ways. For both types of vocalisations as well as for regular echolocation clicks, the times of individual clicks were identified using an automatic click detector with an adjustable threshold. High rate click trains for which individual clicks could not be extracted (7% on average for each individual), due to high flow- or shipping noise, were omitted from further analysis. For those click trains where the click detector detected most of the clicks, the output of the detector was manually inspected and corrected for possible missed or falsely detected clicks.

#### Evaluator classification and examination of acoustic differences

To evaluate the initial classification between high-repetition rate buzzes and the similar high-repetition rate possible calls during auditing, five trained assessors were presented with a subset of 88 initially marked buzzes and 88 initially marked possible calls from each of the six individuals in four-panel figures containing signal envelope, echogram^32,47,50^, ICI and normalised jerk^51^ (see Supplementary Fig. S1). This subset was chosen based on the minimum number of calls emitted from one individual (hp15_218a) to avoid evaluation of the same call twice. Evaluators looked for the presence or absence of prey echoes and rapid changes in acceleration (see Supplementary Material for details). A given click train was considered a buzz if at least one of these two criteria were present, whereas it would be considered a call if neither prey echoes, nor jerk peaks were present. Finally, the classification shared by the majority of evaluators was considered consensus and an inter-evaluator agreement was estimated using Fleiss Kappa^33^.

To examine the acoustic differences between sounds rated by evaluator consensus as buzzes and those rated as calls, the spectrum, received level and repetition rate were quantified for all high-repetition rate click trains. From these, the mean ICI and mean apparent output level (AOL) were quantified. Additionally, the mean AOL of a randomized subset of 10,000 regular clicks (ICI > 15 ms) and of all buzzes and calls, respectively, were quantified from all six individuals to allow for comparison. Mean AOL estimates were obtained by first quantifying the energy flux density (dB re 1µPa^2^·s) as the sum of the pressure squared over the 95% energy duration of each click pulse in a window extending from 100 µsec before to 300 µsec after the peak^3,52^. Finally, individual click energy was averaged over the full click train.

To examine the possibility that clicks used in calls have a different spectral composition compared to regular clicks, a subset of 88 call clicks and 88 regular clicks from each individual were randomly chosen and their estimates of 10^th^, 50^th^ and 90^th^ percentile of centroid frequency, −10 dB bandwidth and −10 dB duration were calculated^52^. This same subset as during initial evaluation was chosen to avoid analysis of several clicks within the same click train.

#### Automatic classification of calls and foraging buzzes

We employed a K-means clustering method^53^ to the subset of 88 buzzes and 88 calls of each individual previously presented to the evaluators, to explore how porpoises potentially may be able to distinguish calls from buzzes and to investigate the existence of possible distinct categories within calls. The appropriate number of clusters for the K-means algorithm was determined by varying the number from two to 10, and examining the silhouette score they generated, as a measure of internal validity. The cluster count generating the highest silhouette score was chosen. Six parameters were used for this classification procedure: duration of the click train and its mean, maximum, minimum, start and end repetition rate. The start and end repetition rate correspond to the median duration between the first six clicks and the six last clicks, respectively. These parameters, thus, are based on the assumption that porpoises only attend to call duration and repetition rate. All parameters were log-transformed and centralised to represent each parameter by equal weight.

#### Co-occurrence of calls of the tagged animal and calls of nearby individuals

If the presumed calls function to mediate social interactions, it is likely that they would often co-occur with calls from nearby untagged animals^54^. The inter-dependence of call rates from the tagged animal and call rates of nearby individuals was therefore investigated. All high-repetition rate click trains initially marked as possible calls and possible conspecific vocalisations were included. First, the total recording time of all deployments was divided into one-minute time bins and the call rate of tagged and untagged individuals quantified within each time bin. From the data, it was found that calls generally occurred in bouts, with autocorrelation coefficients that declined sharply at time lags of five-six minutes, so the average call rate of tagged and nearby animals was calculated over six-minute time bins. As a non-linear measure of how the call rate of the tagged animal tracked the call rate of conspecifics, we calculated the mutual information^55^ using equiquantal binning^56^ of call rates in six-minute time bins. To test if correlations were significantly greater than expected by chance, we used a rotation test^57^ to shift the call rate of conspecifics relative to the tagged animal call rate and measured the time-shifted mutual information. We performed 5000 rotations and calculated the p-value as the proportion of rotations in which the time-shifted mutual information was equal to or greater than the mutual information of the true dataset.

### Data availability

The datasets generated and/or analysed during the current study are available from the corresponding author on reasonable request.

## Electronic supplementary material

Click Communication in Wild Harbour porpoises (phocoena phocoena) - Electronic Supplementary material and figures
