# Supplementary material for: Click communication in wild harbour porpoises (Phocoena phocoena)
Source: Sci Rep. 2018 Jun 26;8:9702. doi: 10.1038/s41598-018-28022-8 (PMC6018799; doi:10.1038/s41598-018-28022-8)
Supplement: Supplementary file 1 — Click Communication in Wild Harbour porpoises (phocoena phocoena) - Electronic Supplementary material and figures [file 41598_2018_28022_MOESM1_ESM.pdf]

Click Communication in Wild Harbour Porpoises (*Phocoena Phocoena*)

Pernille Meyer Sørensen, Danuta Maria Wisniewska, Frants Jensen, Mark Johnson, Jonas Teilmann, and Peter Teglberg Madsen

Electronical Supplementary Material

Electronic Supplementary Material includes a section on the details of experimental procedures, one table and three figures.

Supplemental Experimental Procedures

Study animals and tag deployment

Data were collected through collaboration with local fishermen, who provided access to wild harbour porpoises accidentally caught alive in their pound nets along the coast of Kattegat and the Belt Seas of Denmark. Bycaught porpoises were lifted by hand from the pound net and moved to a soft mat covered by a stretcher on the fishing boat. The tag was attached approximately five cm behind the blowhole, and hence the sound source with four silicone suction cups. In addition to deploying the DTAG the sex of the animal was determined and the length and girth measured before the animal was released back into the water.

Evaluator classification of high-repetition rate click trains as buzzes or calls

To overcome subjectivity in the initial classification of buzzes and calls, expectations concerning foraging buzzes were used to help discriminate them from possible calls. First, most buzzes associated with foraging contain echoes from targeted prey which can be visualised using echograms[1–3]. Second, prey capture should be accompanied by a substantial change in acceleration (i.e., jerk) due to fast contractions of muscles in the gular region[4] to create suction and to grasp the prey[3]. The triaxial jerk (sampled at 250 or 625 Hz) was calculated as the differential of the acceleration in each axis and the total jerk as the norm of the triaxial jerk, estimated by taking the

square-root of the sum of the squared value in each axis (units of $m \cdot s^{-3}$)[5]. Although the presence of echoes and jerk peaks during high rate click trains are objective criteria, it is difficult to establish thresholds for automatic detection as the signal-to-noise ratio of echoes and the movement of the animal change continuously. We therefore used a method of multiple evaluation to classify high-repetition rate click trains as either a buzz or a communication call. A subset of all high-repetition rate click sequences, including one second before the start of the sequence, were presented to five evaluators as a display containing an echogram combined with plots of signal envelope, ICI and normalised jerk as a function of time in a four-panel figure (See supplementary fig. S1). The evaluators had no prior knowledge about the criteria to which the high-repetition rate click trains had been labelled according to, nor did they know how they had been initially labelled. Finally, all evaluators were blind to the answers of other evaluators. In the example of a foraging buzz shown in supplementary fig. S1a, prey echoes are easily recognised and the tail beats of the targeted fish can be seen as intensity variations in the echoes just before the first prey capture attempt 2.5 seconds into the buzz[3]. At the same time a clear jerk peak is generated, which together with the prey echoes indicate that the porpoise is attempting a prey capture. In contrast, a similar plot from a presumed call (See supplementary fig. S1b) reveals no prey echoes and no jerk peaks, suggesting that this click train was not used in the context of foraging despite its high repetition rate. Evaluators were asked to answer "yes" or "no" to two questions based on the presented click sequences: 1) Are any prey echoes present in the echogram? and 2) are any high-jerk peaks present? The five evaluators were asked to go through a randomly selected subset for each animal chosen from sounds initially labelled as possible calls and buzzes. To homogenize the methodology, a sample size of 88 possible calls and 88 possible buzzes from each animal was used, determined by the animal with the fewest number of possible calls (hp15_218a, table 1), giving a total of 1056 high-repetition rate click sequences.

For each high-repetition rate click train, answers of each evaluator were first assessed separately. When neither prey echoes nor jerk peaks were present, the high-repetition rate click train was considered a communication call. All other outcomes were considered buzzes. Thus, any uncertainties would lead to disqualification of the evaluated high-repetition rate click train as a call. Finally, a consensus decision was taken as the classification shared by the majority of the five evaluators (Consensus bar, see supplementary fig. S2) and an inter-evaluator agreement was estimated using Fleiss's Kappa for multiple raters[6], where a score of 1 corresponds to complete

agreement, whereas a score of 0 indicates that the inter-evaluator agreement is no better than that occurring by chance[7].

Table S1. Average inter-click interval (ICI, ms) and average apparent output level (AOL, dB re $1\mu Pa^2\cdot s$) values of all mean ICIs and mean AOLs within all calls and buzzes given for each individual. Additionally, a subset of 10,000 randomly chosen regular echolocation clicks were extracted and the average AOL of all individual clicks for each animal given for comparison. The standard error (SE) for regular echolocation clicks represent individual clicks, whereas SE for calls and buzzes represents mean values of ICI and AOL between individual calls and buzzes.

	**Calls**			**Buzzes**			**Regular echolocation clicks**	
	N	Mean ICI ± SE	Mean AOL ± SE	N	Mean ICI ± SE	Mean AOL ± SE	N	Mean AOL ± SE
hp12_272a	250	12.0±0.33	102±0.52	1857	2.8±0.26	91±0.36		107±0.12
hp12_293a	918	8.3±0.42	106±0.36	1381	3. 1 +0.32	101±0.27		121±0.08
hp13_102a	787	9.9±0.14	111±0.37	3405	3.3±0.54	92±0.35	10,000	112±0.08
hp14_226b	1004	9.3±0.16	106±0.44	3231	3.8±0.46	93±0.34		116±0.06
hp15_218a	88	3.0±0.45	100±0.69	126	3.3±1.12	92±0.80		103±0.09
hp16_264a	657	8.8±0.19	86±0.59	383	3.8±1.19	81±0.41		107±0.08

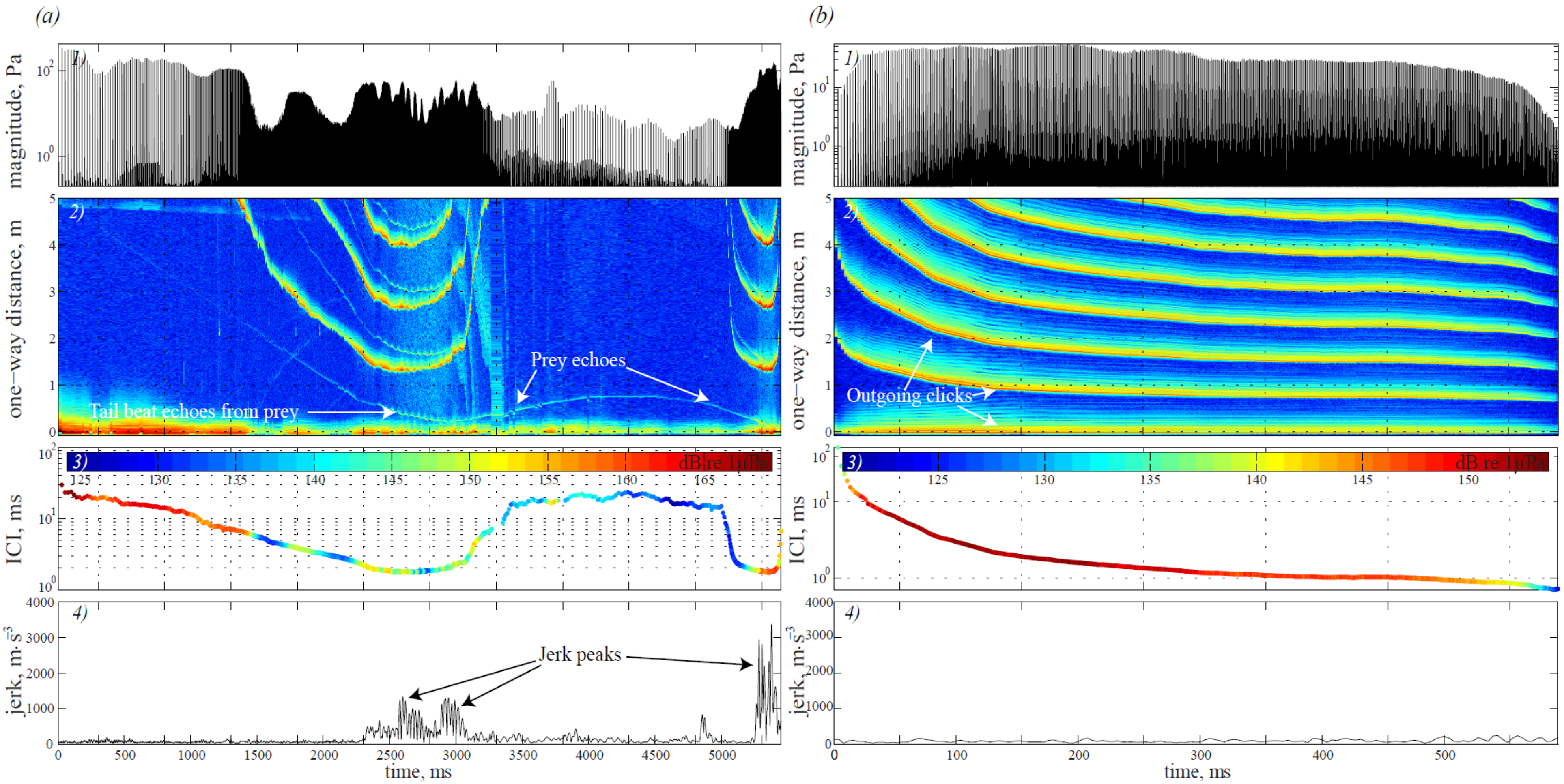

Figure S1. Example click sequences initially labelled as a foraging buzz and a call. For both the buzz (a) and the call (b) examples, the panels represent (from top to bottom): 1) Envelope of the high repetition rate click train. 2) Echogram produced by stacking plots of sound envelopes synchronized to the outgoing clicks. The y-axis displays the time elapsed between the emission of a click and the return of an echo here expressed as range to the object from the sound source assuming a sound speed of 1500 $m{\cdot}s^{-1}$. The clicks emitted at a rate of more than 200 Hz corresponding to an inter-click-interval (ICI) of less than the five-ms window on the y-axis, are repeatedly displayed in the echogram (in (b) labelled as 'outgoing clicks'). In addition to emitted clicks, which can be seen in both (a) and (b), prey echoes and even tail beat echoes from the prey are seen in (a), whereas no prey echoes can be seen in (b). 3) Color-coded inter-click-intervals (ICI) representing variation in apparent output level and the change in repetition rate throughout the click sequence. 4) Norm of jerk, defined as the magnitude of the rate of change in acceleration as recorded on the tag[5]. High jerk peaks have been associated with generation of suction during prey capture.

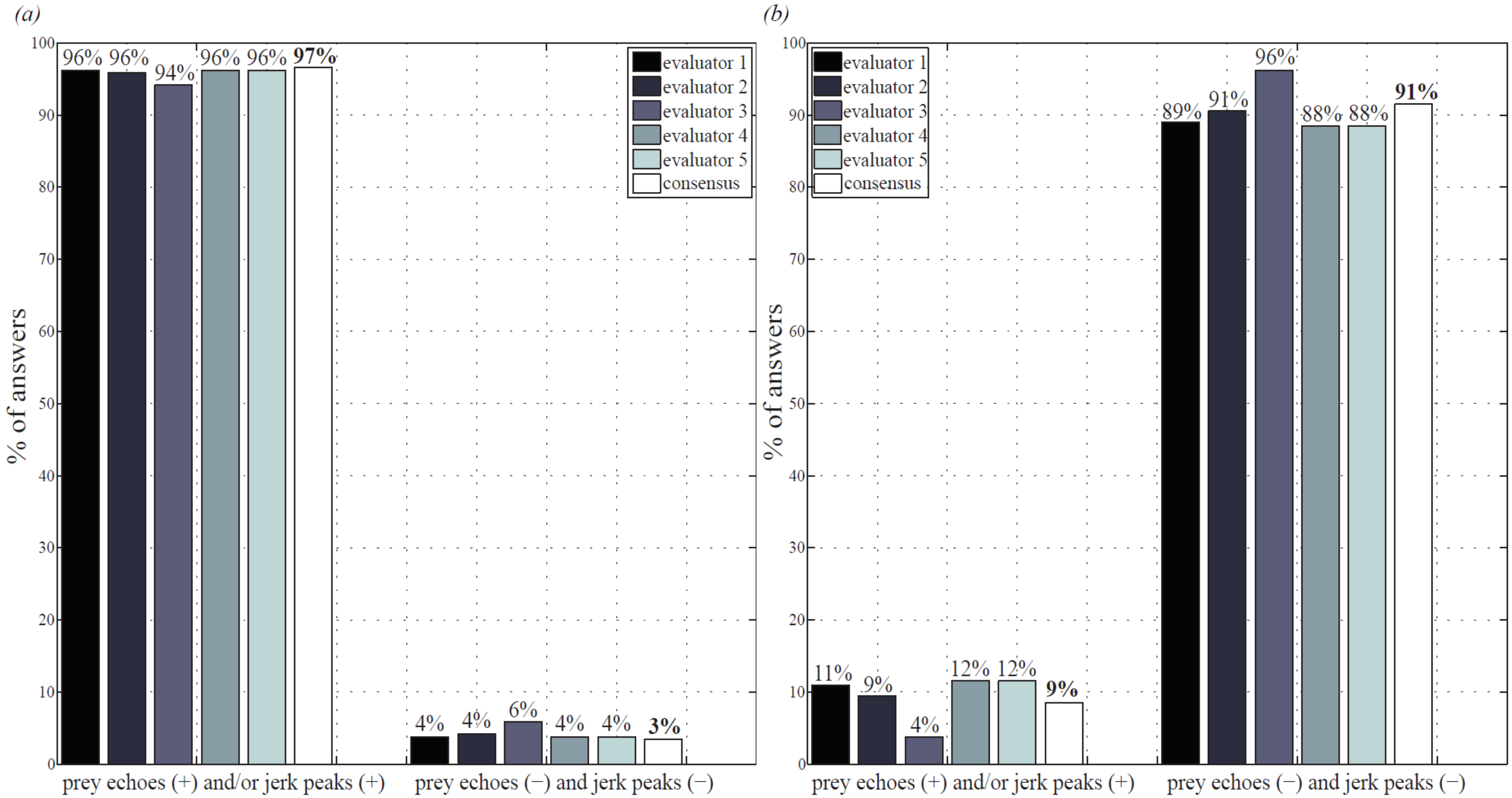

Figure S2. Results of the classification of high-repetition rate click trains as either buzzes or calls by multiple evaluators, based on the associated presence of prey echoes and/or a change in acceleration. Percentage of answers from five evaluators as a function of category for *(*a) high-repetition rate click trains marked as buzzes and *(*b) those marked as possible calls. 'Prey echoes (+) and/or jerk peaks (+)' refers to prey echoes and/or jerk peaks judged to be present in the recording, while 'prey echoes (-) and jerk peaks (-)' refers to neither prey echoes nor jerk peaks present. The bar of consensus represents the merged answers from all evaluators, where the result is that of most votes. For marked buzzes, 97% of all click trains were judged as buzzes, whereas 3% were judged as calls. For marked calls, 91% of all click trains were judged as calls and 9% as buzzes.

68

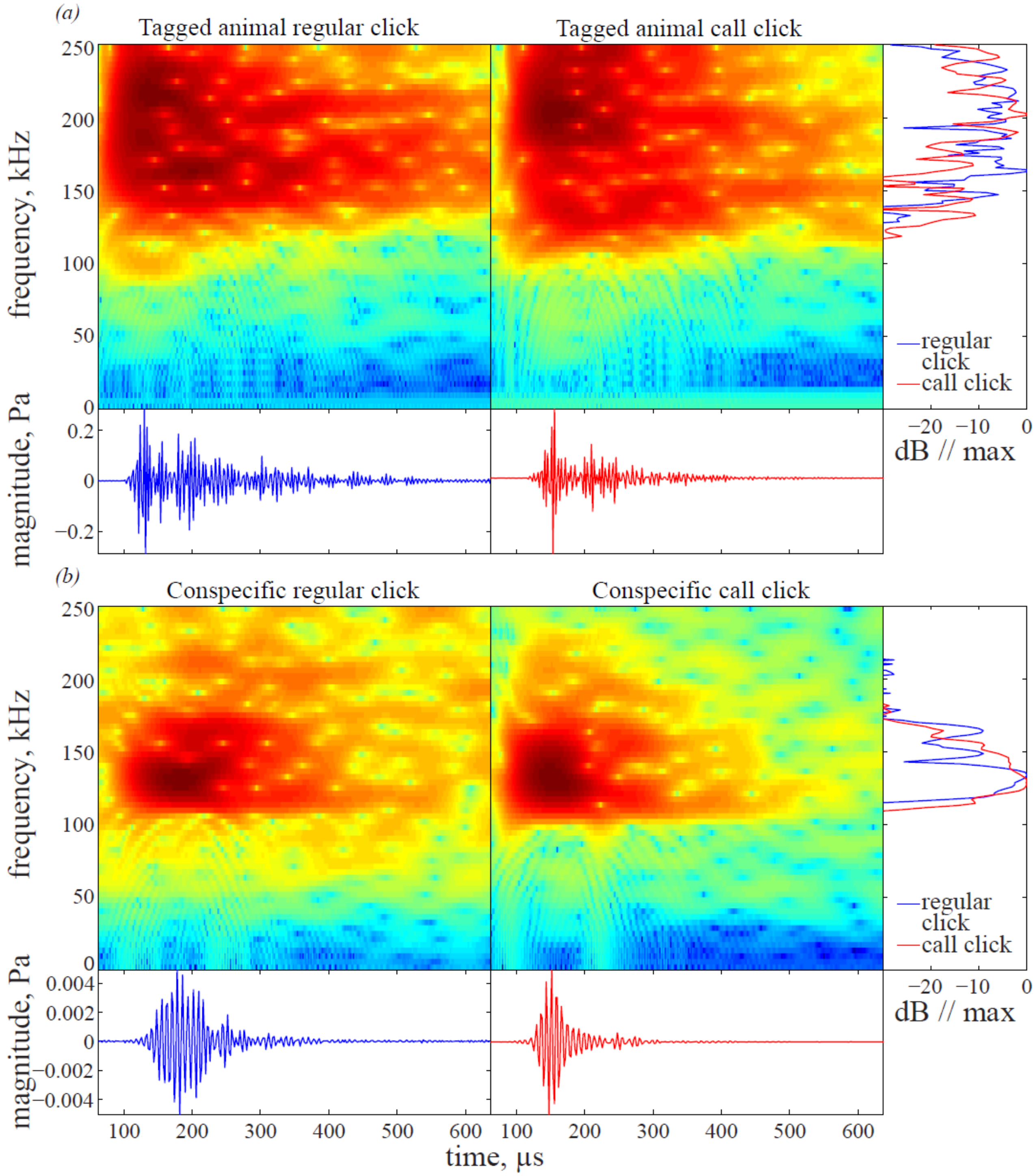

Figure S3. Spectrogram, waveform and spectrum of two representative clicks from regular echolocation and from a call. Spectrum, waveform and spectrum (fft = 128, hanning-window: 64, overlap: 63) of (a) an off-axis click from a regular echolocation click (left, blue) and a possible call click (right, red) from the tagged animal, and (b) click from regular echolocation (left, blue) and from a possible call (right, red) from a nearby conspecific. Both tagged animal clicks were emitted from the same individual (hp12_293a). Note especially the distorted waveform and the high frequency components of the clicks recorded off-axis on the tagged animal compared to the narrow-band clicks recorded on the tag by nearby conspecifics.

69

Supplemental references

1. Johnson, M., Madsen, P. T., Zimmer, W. M. X., Aguilar de Soto, N. & Tyack, P. L. Beaked whales echolocate on prey. *Proc. R. Soc. London. Ser. B Biol. Sci.* **271,** S383–S386 (2004).

2. Johnson, M., Aguilar De Soto, N. & Madsen, P. T. Studying the behaviour and sensory ecology of marine mammals using acoustic recording tags: A review. *Mar. Ecol. Prog. Ser.* **395,** 55–73 (2009).

3. Wisniewska, D. M. M. *et al.* Ultra-High Foraging Rates of Harbor Porpoises Make Them Vulnerable to Anthropogenic Disturbance. *Curr. Biol.* **26,** 1441–1446 (2016).

4. Werth, A. J. in *Feeding: Form, Function and Evolution in Tetrapos Vertebrates* (ed. Schwenk, K.) 475–514 (New York: Academic Press, 2000).

5. Ydesen, K. S. *et al.* What a jerk: prey engulfment revealed by high-rate, super-cranial accelerometry on a harbour seal (Phoca vitulina). *J. Exp. Biol.* **217,** 2239–2243 (2014).

6. Fleiss, J. L. Measuring nominal scale agreement among many raters. *Psychol. Bull.* **76,** 378–382 (1971).

7. Sim, J. & Wright, C. C. The Kappa Statistic in Reliability Studies : Use, Interpretation, and Sample Size Requirements. *Phys. Ther.* **85,** 257–268 (2005).
